# Retraction: The stem cell factor antibody enhances the chemotherapeutic effect of adriamycin on chemoresistant breast cancer cells

**DOI:** 10.1186/1475-2867-13-96

**Published:** 2013-10-18

**Authors:** Neil D Jelly, Issam I Hussain, Jennifer Eremin, Oleg Eremin, Mohamed El-Sheemy

**Affiliations:** 1 University of Lincoln, Brayford Pool, Lincoln LN6 7TS, UK; 2 Research & Development, Lincoln County Hospital, Greetwell Road, Lincoln LN2 5QY, UK; 3 Queens Medical Centre, University of Nottingham, Derby Road Nottingham, Nottingham NG7 2UH, UK

## Retraction

This article has been retracted [1] by the authors because errors were noted in the methodology employed in the experiments which invalidate the results reported. The authors apologise for any inconvenience caused.

## References

[B1] Jelly ND Hussain II Eremin J Eremin O El-Sheemy M The stem cell factor antibody enhances the chemotherapeutic effect of adriamycin on chemoresistant breast cancer cells Cancer Cell Int 2012 12 21 10.1186/1475-2867-12-21 22642642 PMC3413589

